# Directional Analysis of the Storm Surge from Hurricane Sandy 2012, with Applications to Charleston, New Orleans, and the Philippines

**DOI:** 10.1371/journal.pone.0122113

**Published:** 2015-03-30

**Authors:** Carl Drews, Thomas J. Galarneau

**Affiliations:** NCAR Earth System Laboratory, National Center for Atmospheric Research, Boulder, Colorado, United States of America; Coastal Carolina University, UNITED STATES

## Abstract

Hurricane Sandy in late October 2012 drove before it a storm surge that rose to 4.28 meters above mean lower low water at The Battery in lower Manhattan, and flooded the Hugh L. Carey automobile tunnel between Brooklyn and The Battery. This study examines the surge event in New York Harbor using the Weather Research and Forecasting (WRF) atmospheric model and the Coupled-Ocean-Atmosphere-Wave- Sediment Transport / Regional Ocean Modeling System (COAWST/ROMS). We present a new technique using directional analysis to calculate and display maps of a coastline's potential for storm surge; these maps are constructed from wind fields blowing from eight fixed compass directions. This analysis approximates the surge observed during Hurricane Sandy. The directional analysis is then applied to surge events at Charleston, South Carolina, New Orleans, Louisiana, and Tacloban City, the Philippines. Emergency managers could use these directional maps to prepare their cities for an approaching storm, on planning horizons from days to years.

## Introduction

**Fig 1:**
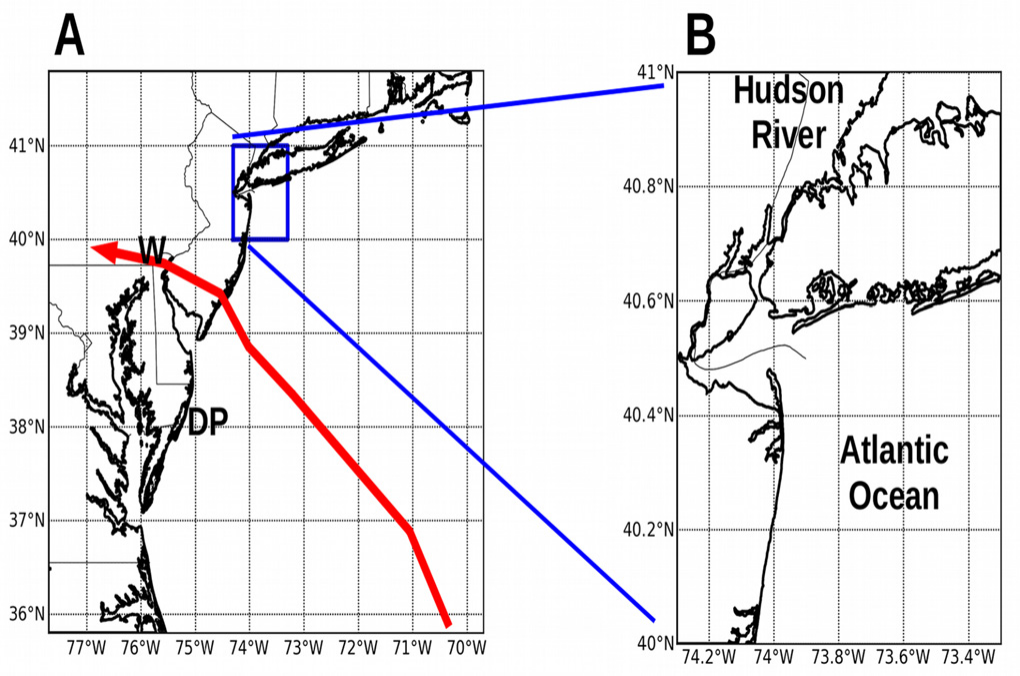
Model domains over the New York City area. Panels: (A) Larger domain centered on the state of New Jersey, with a grid resolution of 824 m and grid size 720 rows x 960 columns. The red line shows the track of Hurricane Sandy 2012.[35] **W** = Wilmington, Delaware; **DP** = Delmarva Peninsula. (B) Smaller high-resolution domain centered over New York Harbor, with grid resolution 82 m and grid size 1200x1200 cells.

New York City is a major metropolitan area located where the Hudson River meets the Atlantic Ocean (see Fig 1). Four of the five city boroughs are on islands clustered around New York Harbor, with bridges and tunnels built to convey traffic across the harbor waterways. The East River provides a tidal connection between New York Harbor and Long Island Sound to the northeast. In nearby New Jersey, the Hackensack River winds through a low-lying marshy area known as the New Jersey Meadowlands, where the Meadowlands Sports Complex was constructed from 1972–2010. Twenty million people live in the New York Metropolitan area.[1]

This study simulates the storm surge in New York Harbor in response to Hurricane Sandy 2012, proposes that a directional analysis can approximate the same surge behavior with a set of fixed wind directions, and then applies that analysis to assess the vulnerability of other coastal cities in the United States and the Philippines.

When a hurricane comes ashore, the wind can come from any angle, depending on the observer's position relative to the eye. During Hurricane Sandy 2012, the storm surge at Kings Point reached a maximum of 3.86 m (without tides) because the wind direction of the approaching storm was aligned with Long Island Sound and its 150 km fetch. (Fetch is the distance of water across which the wind blows.) This alignment may not occur next time; a smaller storm approaching New York Harbor on a track closer to the New Jersey shore may generate winds that blow across Long Island from the southeast, instead of from the east-northeast as in Hurricane Sandy. The magnitude of storm surge is heavily dependent on the **direction** from which the strongest winds are blowing.

### 1.1 Synoptic history of Hurricane Sandy 2012

Tropical storm Sandy became a hurricane at 1200 on 24 October 2012 UTC, about 150 km south of Kingston, Jamaica.[2] It passed over Jamaica, then eastern Cuba and the Bahamas, before embarking on a northeast track roughly parallel to the east coast of the United States and about 480 km offshore through 27 October. The hurricane made a left turn on 28 October and headed toward the southern coast of New Jersey at Category 1 strength. At this point Sandy had grown to an extraordinarily large storm, with tropical-storm-force winds covering an area 1600 km in diameter. The eye came ashore at Brigantine, New Jersey (7 km northeast of Atlantic City) at 2330 on 29 October UTC with winds at 36 m/s (130 km/hr). Hurricane Sandy was reclassified to a post-tropical cyclone just before landfall; the storm dissipated on 31 October over western Pennsylvania.

Other studies have examined the synoptic conditions of Hurricane Sandy and evaluated model predictions. Bassill (2014) and Munsell and Zhang (2014) both examined numerical weather prediction forecasts, with particular emphasis on Sandy's left hook into New Jersey that affected the storm surge.[3][4] Galarneau et al. (2013) provided a synoptic overview of Hurricane Sandy and analyzed factors that contributed to Sandy's pre-landfall intensification and track.[5] They contended that the pre-landfall deepening of Sandy contributed to a ~0.2 m increase in surge near the storm center.

### 1.2 Measurements of wind and storm surge

**Fig 2:**
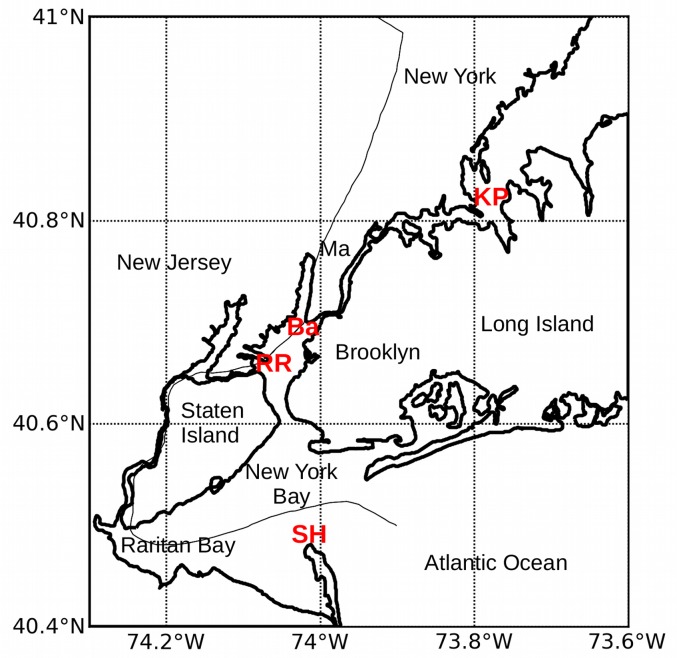
NOAA measuring stations at Kings Point, Long Island, and The Battery, Manhattan, New York. **KP** = Kings Point, Ma = Manhattan Island, **Ba** = The Battery, **RR** = Robbins Reef, **SH** = Sandy Hook. The East River is a tidal waterway that connects the eastern end of Long Island Sound at Kings Point to Upper New York Harbor at The Battery.

The National Oceanic and Atmospheric Administration (NOAA) operates a system of weather stations and tidal gauges for the coastal areas of the United States.[6] This network includes stations around New York Harbor at Kings Point NY (8516945), The Battery NY (8518750), Robbins Reef NJ (8530973), and Sandy Hook NJ (8531680). The map in Fig 2 shows the location of the NOAA measuring stations. Kings Point is located at the western end of Long Island Sound, and The Battery is at the northern end of Upper New York Harbor. Robbins Reef is in the west center of Upper New York Harbor, and Sandy Hook protrudes into Lower New York Bay from the Jersey shore.

The tidal gauge at Sandy Hook ceased operation after 2336 on 29 October UTC, evidently destroyed by the hurricane; the last valid reading was 4.03 meters above mean lower low water (MLLW). The three remaining stations provide verified observations of wind and water levels that we compared with our model results. The average tidal amplitude is about 2.4 m for Kings Point and 1.5 m for The Battery.

Unlike the weather station at The Battery, Robbins Reef is located in the middle of upper New York Harbor, and consequently is able to record winds that are less disturbed by the tall buildings in Lower Manhattan. Robbins Reef recorded a maximum gust of 40.3 m/s at 0024 on 30 October UTC, with a maximum sustained wind speed of 28.3 m/s recorded at 0112 on 30 October UTC. The wind direction at 0112 UTC was 108° from due North, or blowing from 18° south of due East.

**Fig 3:**
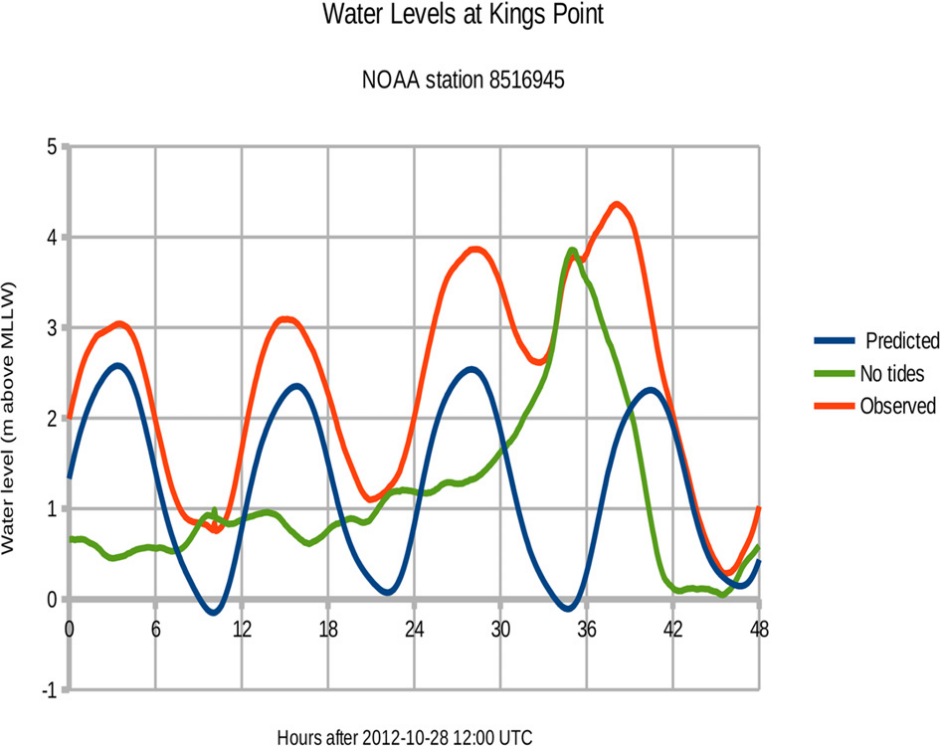
Measured water levels at Kings Point, Long Island. The water level without tides is calculated by subtracting the predicted tidal cycle from the observed water levels. The resulting "No tides" curve represents the storm surge generated by wind stress alone.

The tidal gauge at Kings Point measured a maximum storm surge of 4.36 m above MLLW at 0206 on 30 October UTC. If the predicted tides are subtracted from the measured water level, the resulting curve shows the storm surge due to winds alone (see Fig 3). The maximum wind-driven surge of 3.86 m at Kings Point occurred at 2300 on 29 October 2012 UTC. The peak surge at Kings Point was **not** synchronized with the predicted high tide, which came 5.4 hours later at 0424 UTC on October 30.

**Fig 4:**
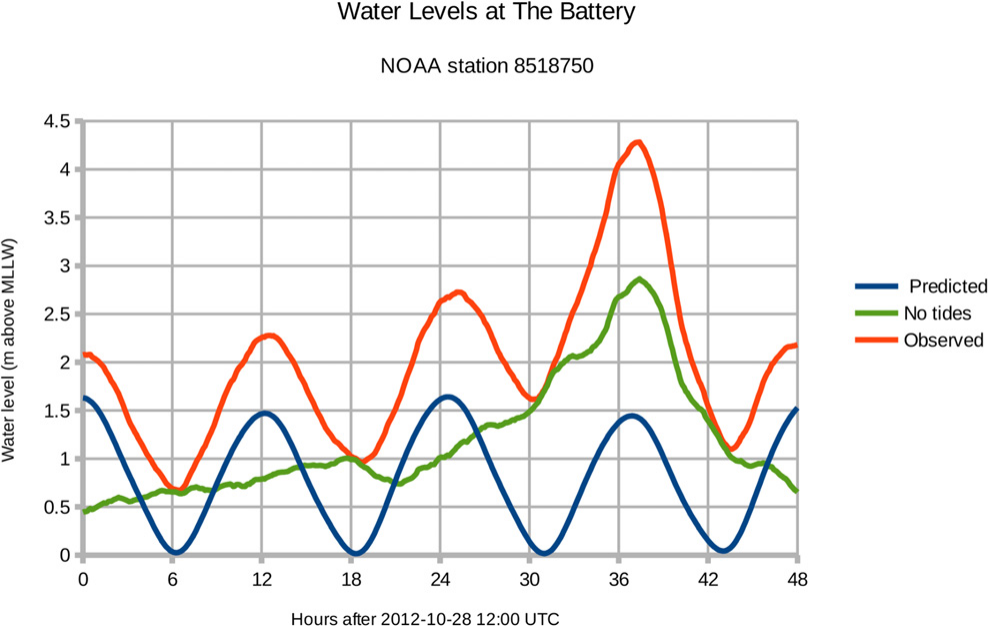
Measured water levels at The Battery, Manhattan. The green "No tides" curve represents the wind-driven storm surge. The total surge during Hurricane Sandy was unusually high in part because the wind-driven component was nearly synchronized with high tide.

The Battery recorded a peak surge of 4.28 m at 0112 on 30 October. This peak **was** nearly synchronized with the predicted high tide, which came only 18 minutes earlier at 0054 UTC (see Fig 4). The adjusted peak surge of 2.87 m at The Battery occurred at 0124 on 30 October 2012. Thus the wind-driven component of the surge peaked 2.4 hours earlier at Kings Point, and was actually 1 meter higher than The Battery.

### 1.3 Damage

The storm surge of Hurricane Sandy brought extensive flooding to the New York City metropolitan area, New Jersey, and Connecticut. Powerful waves also caused shoreline damage in northern New Jersey, Staten Island, and western Long Island. Approximately 305,000 homes were destroyed in New York state alone, mostly by storm surge; 346,000 housing units were damaged or destroyed in New Jersey. Eight transit tunnels were inundated by salt water, including the Hugh L. Carey (Brooklyn-Battery) automobile tunnel between lower Manhattan and Brooklyn. The New York City Metropolitan Transit Authority (MTA) stated that Hurricane Sandy's damage to the subway system was the worst disaster in the system's 108-year history.[2]

147 people lost their lives as a direct result of Hurricane Sandy. Monetary damage to the United States was estimated at $50 billion as of February 2013.[2]

## Methods

Drews (2013) showed that the Coupled Ocean Atmosphere Wave Sediment Transport (COAWST) modeling system can model storm surge accurately on Lake Erie.[7] In addition to the societal importance of analyzing a $50 billion weather disaster, Hurricane Sandy also presents the opportunity to validate those earlier results under more severe weather conditions. For an operational forecast system, Drews (2013) recommended adjusting the wind stress by a factor of **1.53** when waves are not included in the ROMS (Regional Ocean Modeling System) component of COAWST. The Lake Erie study calculated an increase in setdown/surge of **6.5%** (average of 7.6% and 5.5%) from 2-D to 3-D operation in ROMS. For the Hurricane Sandy experiments we ran COAWST/ROMS in its two-dimensional configuration, without waves, and multiplied the wind stress by a combined adjustment factor of **1.63** (= 1.53 x 1.065).

Benchmark tests reveal the operational advantage in running a simpler model configuration. The 2-D configuration of COAWST took 4.5 wall-clock hours to run 24 simulated hours on a Dell Optiplex 960 workstation. The equivalent 3-D configuration took 90 wall-clock hours to run, or 20 times as long, using three vertical levels and ten 2-D timesteps for each 3-D step. The 3-D configuration with waves took 253 hours, or 56 times as long, using two processors for ROMS and SWAN. Although a supercomputer would run these ocean models much faster, any real-time forecasting system would be under considerable pressure to produce model output in time for emergency managers to take action. A simpler 2-D model configuration is advantageous if the calculated water levels match the 3-D versions. The numerical results are compared in experiments C1, C2, and C3.

### 2.1 WRF atmospheric model

The numerical simulation of Hurricane Sandy was generated using version 3.4.1 of the Advanced Research WRF (ARW) model.[8] The simulation was made over an outer domain at 15 km horizontal resolution that covered much of North America and the western North Atlantic ocean basin. A two-way inner nest of 3 km was employed that covered a region extending from the southwest Gulf of Mexico on the lower left to near eastern Newfoundland, Canada, on the upper right. The simulation used 36 vertical levels extending up to 20 hPa.

The initial and lateral boundary conditions were provided by the National Centers for Environmental Prediction (NCEP) Global Forecast System (GFS) analyses, available on a 0.5x0.5 degree latitude-longitude grid, with the lateral boundary conditions updated every 6 hours. Most of the physical parameterizations used are similar to those often used in real-time hurricane prediction using the ARW model.[9] These parameterizations include: Yonsei University boundary layer,[10] WRF single-moment 6-class microphysics scheme,[11] rapid radiative transfer model for longwave and shortwave radiation (RRTMG), Noah land surface,[12] Hybrid Coordinate Ocean Model (HYCOM) ocean analysis for ocean mixed-layer depth and sea surface temperature, second order diffusion, positive definite scalar advection, and Smagorinsky turbulence. The Tiedtke cumulus parameterization[13] is used on the 15 km domain, with explicit convection on the 3 km inner nest.

**Fig 5:**
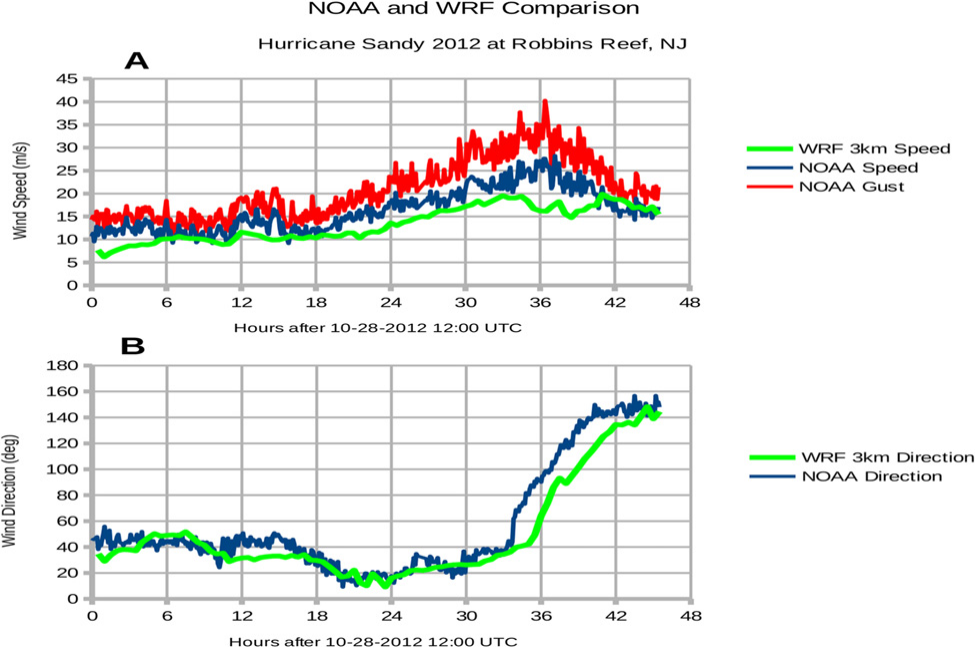
Modeled and measured wind at Robbins Reef, New Jersey. Panels: (A) wind speed, (B) wind direction, blowing from degrees clockwise as measured from due North. The WRF atmospheric model provides wind speed and direction at a height of 10 m and grid resolution 3 km at 30-minute intervals. Actual wind measurements are retrieved from the NOAA weather station at Robbins Reef (8530973) at 6-minute intervals.

Fig 5 shows a comparison of the 10 m wind speed at Robbins Reef, New Jersey, between the WRF model simulation and observations. The observed wind speeds peaked near ~28 m s-1 (with gusts over 40 m s-1) around 0000 UTC 30 October 2012 (Panel 5A). The WRF simulation was characterized by a reduced peak near 20 m s-1 that occurred nearly 3 hours earlier than in the observations. The wind direction in the WRF simulation compared well with observations overall, except that winds began to veer from northeasterly to southerly nearly 3 hours later compared to observations (Panel 5B). This difference in wind direction is due to the relatively faster northwestward progression of Sandy in the WRF simulation. While the wind speed is underdone and the direction is too northerly after 2100 UTC 29 October, the track of Sandy was well represented by the WRF simulation and offers a reasonable case to test the storm surge prediction capabilities of COAWST/ROMS.

The 10-m wind speed was somewhat underestimated in the WRF simulation. At about 30 hours, just prior to landfall, the 10-m wind speeds were 3–4 m/s too weak. This low error, however, is well within the bounds of typical intensity errors from real-time 24–36 hour forecasts as shown by Rappaport et al.[14](their Fig 6). The increase in 10-m wind speed error after 30 hours may be related to Sandy's landfall over New Jersey and rapid weakening in the WRF simulation, which occurred approximately 3 h earlier than observed. A more detailed diagnosis of the intensity forecast errors for Sandy is a good subject for a separate study.

**Fig 6:**
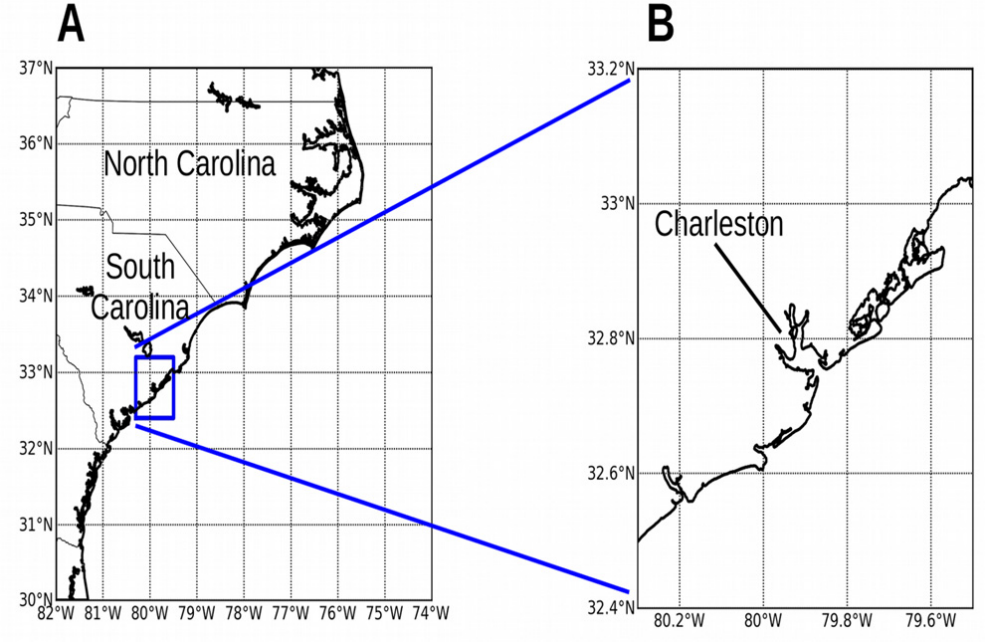
ROMS model domains over the Carolinas and Charleston. Panels: (A) Larger domain centered on the states of North and South Carolina, with a grid resolution of 850 m and grid size 840 rows x 960 columns. (B) Smaller high-resolution domain centered over Charleston, South Carolina, with grid resolution 85 m and grid size 960x960 cells.

### 2.2 COAWST ocean model

COAWST is a coupled modeling framework that can exchange data fields between the ocean model ROMS, the atmosphere model WRF, the wave model SWAN, and the Community Sediment Transport Model.[15] In this study the wind fields of WRF supply one-way forcing for the COAWST ocean model; WRF and ROMS are **not** coupled. WRF calculates the west-east (**u**) and south-north (**v**) wind components at 10 meters above sea level and outputs these values every 30 minutes from 12:00 28-Oct-2012 UTC until 09:30 30-Oct-2012 (total 45.5 hours). The wind speed is converted to surface stress using the formulation given by Oey et al. (2006).[16] WRF also supplies barometric pressure forcing at sea level. The 3 km WRF output grid is converted into a pair of COAWST domains at 800 m and 80 m grid resolution using linear interpolation.

The COAWST algorithm for wetting and drying is enabled. This algorithm uses "critical depth" as the minimum calculated depth for water in a grid cell. The critical depth for ROMS (version 3.4) is set to 0.5 meters, and the time step for calculation is 2.0 seconds for both domains. Since the standard two-dimensional configuration of COAWST does not include barometric pressure forcing, we duplicated the Fortran code that reads the atmospheric pressure field from the 3-D version into the 2-D configuration. The quadratic bottom drag coefficient (RDRG2) is 1.0e-3, as recommended by Drews (2013).[7] The ocean model does not include tides.

The 800 m outer domains use no-slip walls for their lateral boundaries, and begin from a state of no motion. Closed outer boundaries are appropriate for the directional analysis because the domain boundaries are a long distance from the coast (~100 km), the forecast periods are short (24 hours), and we see no boundary effects near the coastal region of interest. The inner domains do not provide a long enough fetch distance to model storm surge accurately, and consequently must be forced from the outer domains (as in Fig 1). The 800 m New Jersey domain runs first and generates lateral boundary conditions for the 80 m New York Harbor domain; the inner domain uses Chapman[17] for the free surface elevation, and Flather[18] for the boundary currents.

### 2.3 Directional analysis

The purpose of the directional analysis is to isolate the effect of wind direction on storm surge. A simulated wind blows across the domain at 8 different angles representing the compass directions, using an increment of 45° between cases (0°, 45°, 90°, 135°, etc.). The wind speed increases from 0 m/s to 46 m/s over 24 hours; this linear ramp approximates the right-hand side of a Category 2 hurricane coming ashore (northern hemisphere). The right-side landfall is generally recognized to be the worst case for storm surge. At the end of 24 simulated hours, COAWST records the height of the water's free surface (the variable *zeta)* across the entire domain. This ending snapshot represents the **surge potential** of that coastline for hurricane-force winds blowing from a specific direction.

The Coriolis force deflects ocean currents to the right in the northern hemisphere, and to the left in the southern hemisphere. Since the directional analysis is intended to model the realistic response to a specific wind direction, the Coriolis force is determined by latitude as usual. The directional analysis does not include tidal effects, but instead calculates results that are independent of the tidal cycle.

Hurricane Katrina 2005 came ashore at the Louisiana/Mississippi border on August 29 as a large Category 3 hurricane. At this final landfall the hurricane's eye was approaching the Louisiana coast from the south, and an untrained observer in New Orleans might therefore conclude that the storm surge would also come from the **south**. In reality, floodwalls adjacent to the 17^th^ Street Canal were breached by a storm surge of 3–4.3 m coming from the **north**, and impacting the southern shores of Lake Pontchartrain. Eighty percent of the city of New Orleans flooded to varying depths up to about 6.1 m.[19] A directional analysis should have revealed the danger to New Orleans of storm surge coming from the north.

### 2.4 Cases: New York, Charleston, New Orleans, Tacloban City, Tanis

The large model domains are created using topographical data from the Shuttle Radar Topography Mission at a horizontal resolution of 30 arc-seconds (SRTM30), and the inner high-resolution domains are created at 3 arc-seconds (SRTM3).[20] Thus the grid cells are approximately 800 m and 80 m wide. Table 1 shows the latitude and longitude bounds for each domain. We retrieved bathymetry data at 30 arc-seconds from NOAA's Environmental Research Division's Data Access Program (ERDDAP),[21] and merged it at the waterline with SRTM3 data above sea level at 3 arc-seconds to create a high-resolution 80 m terrain model.

**Table 1:** Domain boundaries.

Domain	Latitude 1	Longitude 1	Latitude 2	Longitude 2
New Jersey	35.8° N	77.7° W	41.8° N	69.7° W
New York City	40.0° N	74.3° W	41.0° N	73.3° W
Carolina	30.0° N	82.0° W	37.0° N	74.0° W
Charleston	32.4° N	80.3° W	33.2° N	79.5° W
Louisiana	26.0° N	90.7° W	30.8° N	85.0° W
New Orleans	29.6° N	90.5° W	30.4° N	89.6° W
Visayas	7.0° N	121.0° E	15.0° N	129.0° E
Tacloban City	10.8° N	124.6° E	11.7° N	125.4° E
Tanis	30.5° N	31.5° E	31.5° N	33.0° E

The model domains are listed in pairs, with the outer domain first. The outer domain has a grid resolution of 800 m, and the inner high-resolution domain has a resolution of 80 m. All coordinates are in decimal degrees of latitude and longitude. The first coordinate pair specifies the southwest corner, and the second coordinate pair refers to the northeast corner. The Tanis domain is 80 m and has no outer domain.

The SRTM3 data for New York Harbor contains some data errors that affect coastal modeling. The topography shows a hole 86 m deep at 40.6958° North, 74.1617° West, which is one of the runways at Newark Airport. This geographical anomaly causes numerical instability in the ocean model and must be corrected.

The bathymetry data at 800 m cannot fully resolve many of the channels in New York Harbor. For example, the Hudson River is reported by SRTM30 to have a depth of 0.1 meters between The Battery and Chelsea Waterside Park (latitude 40.70° N to 40.75° N). The East River between the Brooklyn Bridge and the eastern end of Randall's Island has a reported depth of 0.1 meters. We considered these connections to be important for our study of storm surge, expecting that there would be significant flow along the East River between Long Island Sound and upper New York Harbor during certain phases of Hurricane Sandy.

#### 2.4.1 Terrain Modifications to New York Harbor

In order to simulate the channel flow, we modified the raw SRTM30 bathymetry based on NOAA's nautical charts giving the channel depth for the Hudson and East Rivers.[22] The modeled Hudson River at mid-channel along lower Manhattan is 15 meters deep. The depth of our digital East River is:

- 19.8 meters from Rikers Island through Hell Gate to Mill Rock.
- 8.53 meters east of Roosevelt Island
- 15.24 meters west of Roosevelt Island
- 12.2 meters from Roosevelt Island to Governors Island

The channel width was chosen to match Google Earth imagery.

The original topography at 80 m grid resolution was modified using the same techniques developed by Drews and Han (2010).[23] We draw a path along the visible river channel with Google Earth, imported the path coordinates as (latitude, longitude) into a Python script, converted them into (row, column) grid coordinates for the model domain, and then increased the bathymetry depth to match the average value given on the nautical chart. The channel profile is an ellipse. We used a Google Earth polygon and a flood-filling algorithm in a similar manner to correct the data error at Newark Airport. S1 File contains the channel modifications in KML format suitable for viewing with Google Earth.

The terrain modifications can be thought of as "digital dredging" of the harbor channels. The channels around Staten Island were digitally dredged to a depth of 14 meters for the Kill van Kull north of the island, and 11 meters for the Arthur Kill to the west. The Harlem River was increased in depth to 5.5 meters around the east and north of Manhattan Island, although at 130 meters wide this channel cannot be well-resolved at a grid resolution of 80 m.

#### 2.4.2 Other Model Domains

We modified the SRTM3 topography of Lake Pontchartrain to obtain accurate bathymetry within the lake. The average depth of Lake Pontchartrain is about 4 m.[24] Since the SRTM3 data shows a shallower lake, we modified Lake Pontchartrain to have a uniform depth of 4 m, using Google Earth and the flood-fill algorithm described in Section 2.4.1.

The Lake Pontchartrain Causeway crosses the center of the lake from Mandeville to Metairie; Interstate 10 and Highway 11 cross the eastern end of the lake, alongside the Norfolk Southern Lake Pontchartrain Railway Bridge. These bridges are represented in the SRTM3 data as elevated solid causeways that would block water flow completely. However, these roadways are actually elevated on **piers** that permit water to flow freely beneath the bridge surface. We removed the causeway and bridges by setting their depth to that of the surrounding lake bottom.

**Fig 7:**
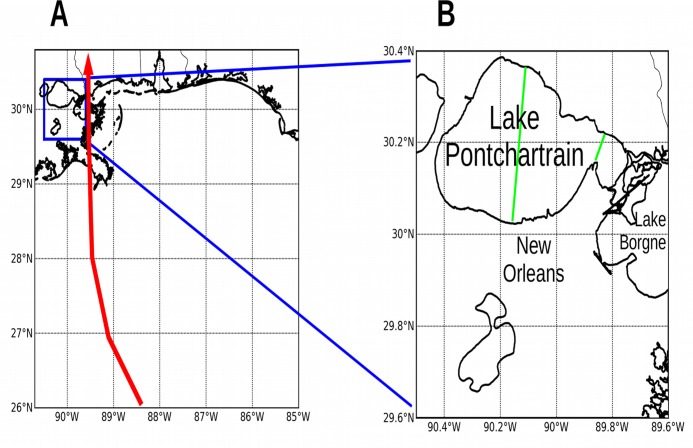
ROMS model domains over New Orleans and Louisiana. Panels: (A) Larger domain centered on the Gulf coasts of Louisiana, Mississippi, Alabama, and Florida, with a grid resolution of 870 m and grid size 576 rows by 684 columns. The red line shows the track of Hurricane Katrina 2005.[35] (B) Smaller high-resolution domain centered over New Orleans with grid resolution 86 m and grid size 960x1080 cells. Green = bridges removed.

**Fig 8:**
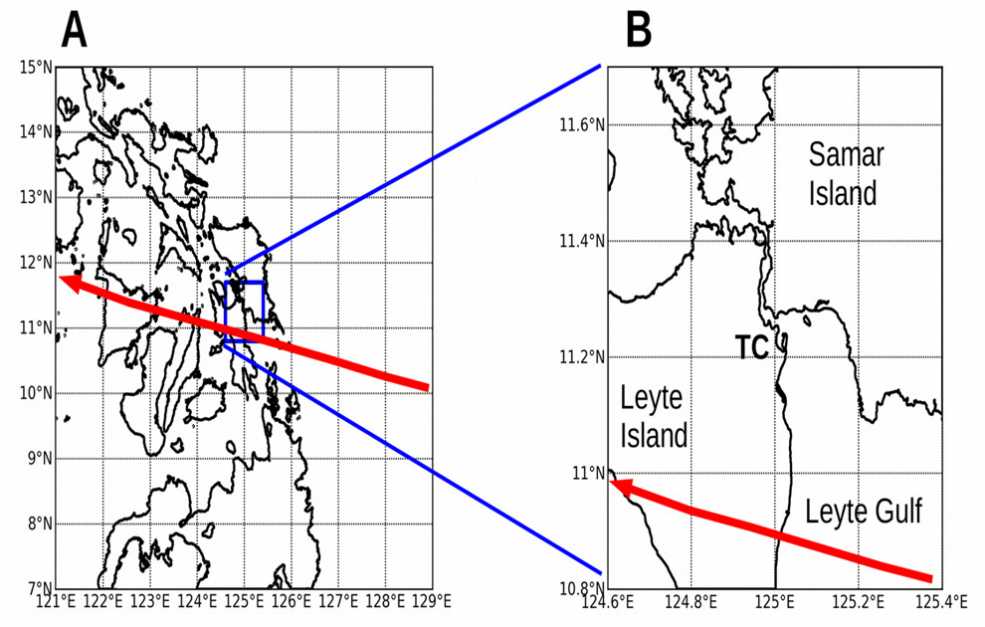
ROMS model domains over Visayas region, the Philippines, and Tacloban City. Panels: (A) Larger domain centered on the central Philippines, with a grid resolution of 918 m and grid size 960 rows x 960 columns. (B) Smaller high-resolution domain centered over Tacloban City (**TC**), with grid resolution 92 m and grid size 1080x960 cells. The red lines show the track of Typhoon Haiyan 2013.[36]

The topography of the other model domains was kept as retrieved from the Shuttle Radar Topography Mission, and was not modified. Fig 6 shows the nested model domains at Charleston, South Carolina. Fig 7 shows the model domains at New Orleans, Louisiana. Fig 8 shows the model domains at Tacloban City, Visayas region, the Philippines.

### 2.5 Model Experiments

Two historical storms provided input to the COAWST surge model. We simulated Hurricane Sandy 2012 over the New York / New Jersey domains, using 10-m wind fields generated by WRF (Section 2.1). Super Typhoon Haiyan in November 2013 reached a maximum intensity of Category 5 in the western Pacific; to approximate the effects of this storm on the central Philippines we used a wind speed of 70 m/s blowing from the southeast, ramping up over 24 hours as in the directional analysis. The directional analysis ran on all eight model domains, using the outer domain as boundary forcing for the inner domain. Table 2 shows the model experiments. Model results are available for download at the Earth System Grid, and may be retrieved through the NCAR Gateway by registering there.[25]

**Table 2:** Model experiments.

Domain	Hurricane	Directional
New Jersey	NJ5	NJ7, NJ8
New York City	NY6, NY9	NY7
Carolina		C1, C2, C3
Charleston		Ch3
Louisiana		L1
New Orleans		NO1, NO5
Visayas		V1, V2
Tacloban City		TC1, TC2
Tanis		T22

The Hurricane column shows experiments that simulate a historical storm. The Directional analysis runs an idealized wind from all eight points of the compass, using an increment of 45°. The outer domain forces the inner; for example, the surface height and currents generated by C1 are used as boundary conditions for Ch3. Special experiments: NY9 has the East River **not** dredged; NJ8 does **not** include the Coriolis force; T22 uses a degree increment of 5°.

## Results

We focused our attention on the **height and timing of the maximum surge** reached during Hurricane Sandy, on the grounds that this maximum value would pose the most danger to the infrastructure around New York Harbor. The water level's behavior during the rising and falling phases was of secondary importance. Although it would be desirable for the modeled water level to match observations closely over the entire time series, this study emphasizes the highest surge reached and its occurrence in time. Any emergency flood barriers would have to be fully in place by the forecast time of the peak surge.

### 3.1 Hurricane-driven storm surge

**Fig 9:**
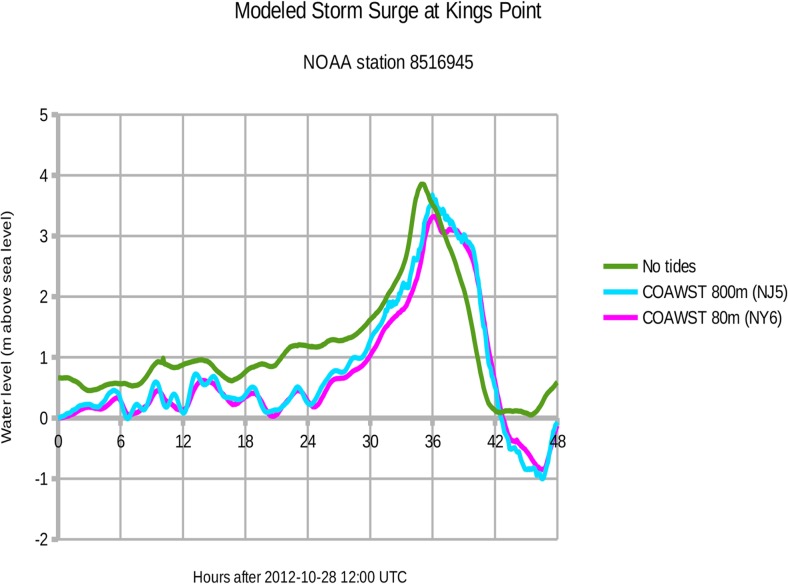
Storm surge at Kings Point during Hurricane Sandy 2012. Green = observed water level with natural tides subtracted. Cyan = COAWST model output for the 800 m New Jersey domain (experiment NJ5). Magenta = COAWST output for the 80 m New York City domain (experiment NY6).

Fig 9 shows the observed and modeled water levels at **Kings Point** measuring station 8516945. The observed peak surge of 3.86 meters above sea level occurred at 2012-10-29 23:00 UTC (tides removed). For the 800 m New Jersey domain (experiment NJ5), the modeled peak surge of 3.68 m occurred at 2012-10-30 00:00 UTC. Thus the modeled peak surge is 18 cm below observations (95% of observed) and 1.0 hours later for NJ5. For the 80 m New York domain (experiment NY6), the modeled peak surge of 3.32 m occurred at 2012-10-30 00:06 UTC. This modeled peak surge is 54 cm below observations (86%) and 1.1 hours later for NY6.

**Fig 10:**
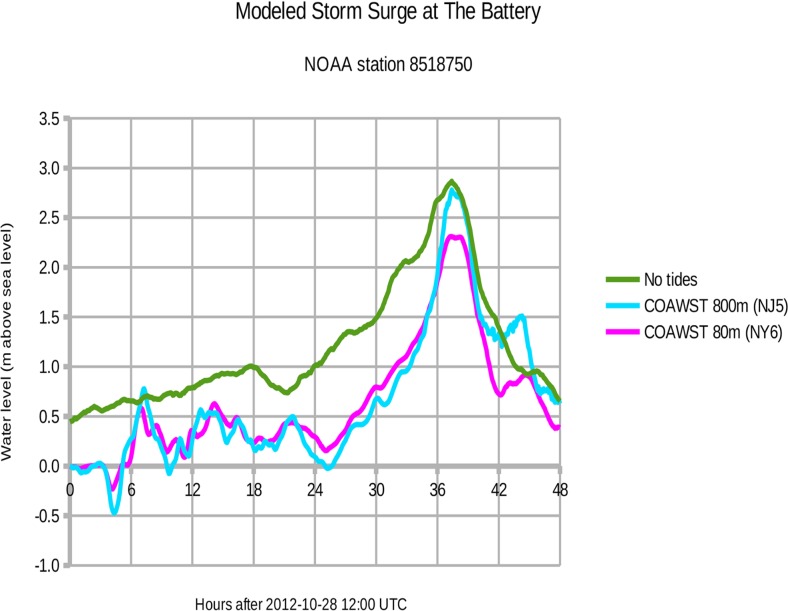
Storm surge at The Battery, Manhattan, during Hurricane Sandy 2012. Green = observed water level with natural tides subtracted. Cyan = COAWST model output for the 800 m New Jersey domain (experiment NJ5). Magenta = COAWST output for the 80 m New York City domain (experiment NY6).

Fig 10 shows the observed and modeled water levels at **The Battery**, lower Manhattan. At measuring station 8518750, the observed peak surge of 2.87 meters above sea level occurred at 2012-10-30 01:24 UTC (tides removed). For the 800 m New Jersey domain (experiment NJ5), the modeled peak surge of 2.78 m occurred at 2012-10-30 01:24 UTC. Thus the modeled peak surge is 9 cm below observations (97% of observed) and exactly on time for NJ5. For the 80 m New York domain (experiment NY6), the modeled peak surge of 2.31 m occurred at 2012-10-30 01:24 UTC. This modeled peak surge is 56 cm below observations (80%) and exactly on time for NY6.

Fig 10 shows a consistent underestimation of the observed surge, and we attribute this difference to the underestimation of observed wind speed shown in Fig 5. Fig 10 also reveals that the outer 800 m domain exhibits more extreme water levels, especially during the peak surge at 37.4 hours (2012-10-30 01:24 UTC). In confined spaces like Upper New York Harbor and eastern Raritan Bay, the 800 m domain cannot resolve narrow channels such as the Hudson River. Consequently, the wind-driven water is unable to flow away from The Battery and therefore the modeled surge is greater there by about 0.5 m. Along more exposed shorelines like the Jersey Shore, the modeled surges for the 800 m and 80 m domain agree to within 0.1 m. Note that the SLOSH model is accurate to +/- 20%.[26]

Experiment NY9 ran Hurricane Sandy without the East River channel. Peak surge at Kings Point was 0.2 m higher than NY6 (about 6%).

### 3.2 Directional surge

#### New Jersey / New York City

Experiment NJ7 represents the standard directional analysis performed on the (outer) New Jersey domain with Coriolis parameter *f* = 9.14e-5. Experiment NJ8 represents the directional analysis with the Coriolis parameter set to 0. The difference illustrates the importance of the Coriolis force in the outer 800 m domains. At Stafford Township, New Jersey, a wind blowing at 46 m/s from due north produces a storm **surge** of 0.7 meters with Coriolis included, and a wind **setdown** of 0.9 meters without Coriolis. (Wind setdown is the drop in water level.) The sea level rises with Coriolis because Ekman transport directs the southward flow toward the Jersey shore. All subsequent directional analyses were performed with Coriolis in order to calculate the actual ocean response according to the wind direction.

In experiment NJ7 the maximum surge at Kings Point is 6.5 m with wind blowing from the east. The maximum surge at The Battery is 5.0 m with wind blowing from the southeast. These results are about 1.7 times greater than the actual surge measured during Hurricane Sandy: 3.86 m at Kings Point, and 2.87 m at The Battery, with tides removed. The higher surge is explained by noting that the directional analysis uses a Category 2 hurricane, while Sandy was downgraded from a Category 1 just before landfall.

The directional analysis illustrates that Kings Point and The Battery are vulnerable to storm surge driven by winds from different directions, which in turn depend on the track and size of the approaching hurricane. However, the most vulnerable portion of the nearby coastline is shown to be the Delaware estuary. At Wilmington the water rises to **8.7 m** when winds at Category 2 strength are blowing from the southeast. This situation would roughly correspond to a hurricane on a northwesterly track making landfall on the central Delmarva Peninsula. The long shallow estuaries of the North American east coast generate higher storm surge than relatively smaller inlets like New York Harbor.

Experiment NY7 uses the outer domain NJ7 to boundary-force the inner 80 m domain centered on New York City. The maximum surge at Kings Point is 6.2 m with wind blowing from the east, and the maximum surge at The Battery is 4.6 m with wind blowing from the southeast. These values average out to 94% of the NJ7 values; the surge is smaller in NY7 because the 80 m domain can resolve the East River and Hudson River channels, thereby allowing peak surge to flow away from these measuring stations in the high-resolution COAWST model.

**Fig 11:**
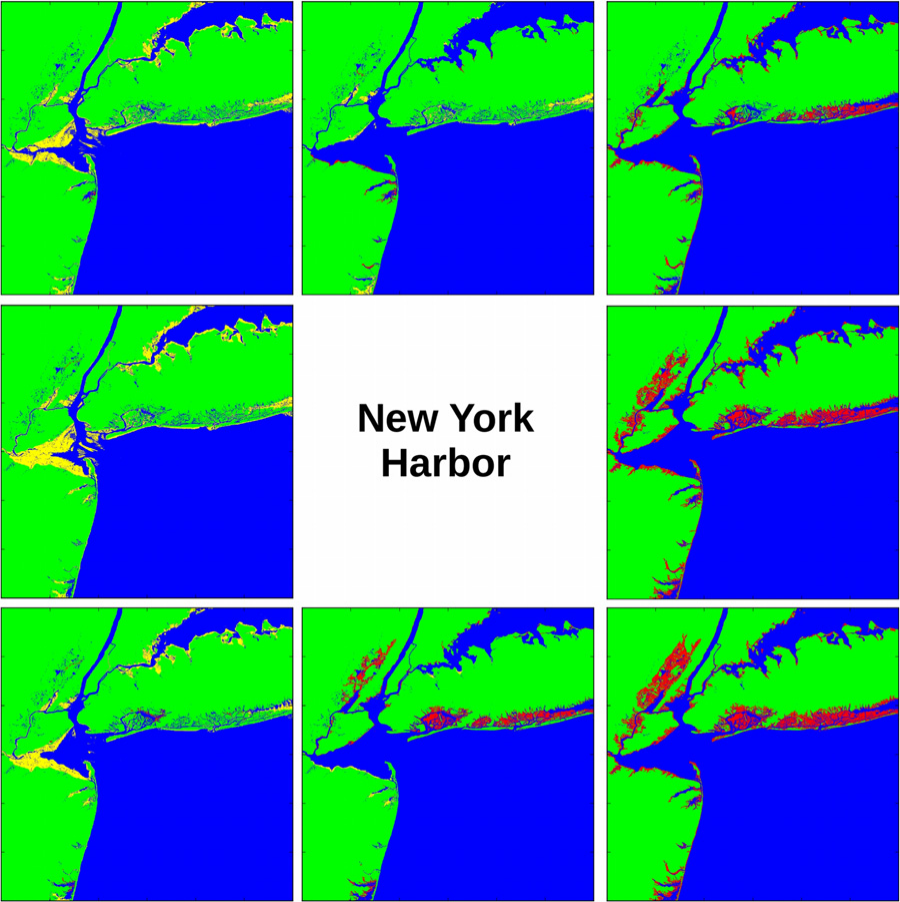
Directional analysis at New York Harbor (experiment NY7). Wind blows toward the center of the figure, like a wind rose diagram. The eight panels display the storm surge and wind setdown that results from wind out of that compass direction. Colors: green = land surface, blue = sea, yellow = sea floor that has blown dry, red = land surface that has been flooded.

**Fig 12:**
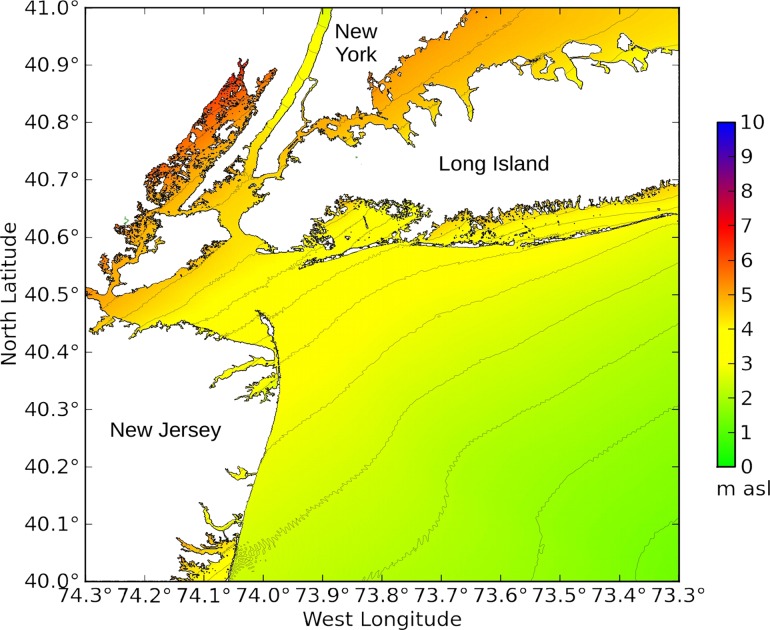
New York Harbor under winds blowing from the southeast (experiment NY7). This plot displays a more detailed view of the lower right-hand panel in Fig 11. The color scale shows storm surge height in meters above sea level.

The directional analysis may be visualized by creating a set of maps similar to a wind rose. In Fig 11 the surge plots for eight compass points are arranged around the center, with the wind direction understood to be blowing **toward** the center of the composite plot. This diagram would be implemented on a web site by allowing the user to click on a single panel and bring up the high-resolution version shown in Fig 12 for wind blowing out of the southeast. In this way forecasters could use preliminary forecasts of the hurricane track to estimate the most vulnerable areas of coastline. For example, the right side of a hurricane impacting the U.S. east coast will be more susceptible to storm surge because the wind force is directed **on**-shore.

#### Carolina / Charleston

Model experiment C1 represents the directional analysis of the outer Carolina domain, covering the Atlantic coasts of North Carolina, South Carolina, and Georgia. Experiment Ch3 represents the directional analysis at the city of Charleston, South Carolina, using the output of experiment C1 as boundary forcing.

**Fig 13:**
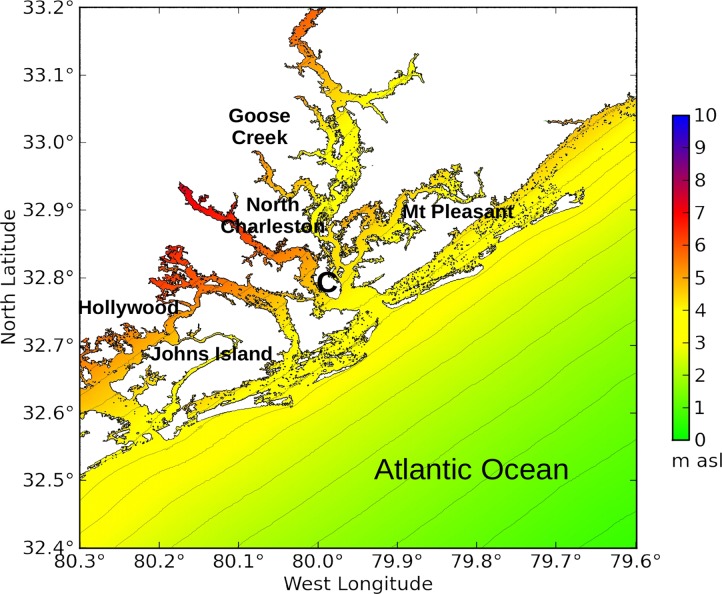
Charleston Harbor under winds blowing from the southeast (experiment Ch3). **C** = downtown Charleston. The color scale shows storm surge height in meters above sea level.

Fig 13 shows the surge at Charleston, South Carolina, resulting from wind speed increasing to 46 m/s from the southeast. Storm surge rises to 3.6 m at the outer entrance to Charleston Harbor between Morris Island and Sullivan's Island, and 4.1 m at Battery Park in south Charleston City. Flooding along the inland waterways is considerably higher than along the Atlantic shoreline. For example, the surge under Limehouse Bridge at the north end of Johns Island reaches 5.5 m above sea level. Although these values are much smaller than the extreme surge at Wilmington, Delaware, the directional analysis shows that Charleston Harbor is vulnerable to a hurricane making landfall about 50 km south of the city.

#### Louisiana / New Orleans

**Fig 14:**
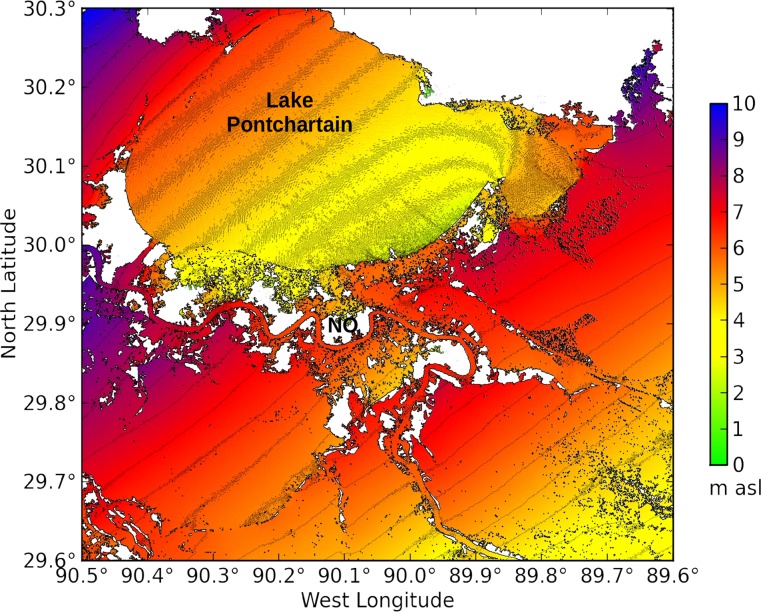
New Orleans under winds blowing from the southeast (experiment NO1). **NO** = downtown New Orleans at the center of the figure. The color scale extends to 10 m above sea level.

The directional analysis NO1 shows widespread flooding across the greater New Orleans region under a Category 2 wind blowing from the southeast (Fig 14). Although surge heights of greater than 6 meters may seem extreme, recall that Hurricane Katrina generated surge heights of **8.47 m** at Pass Christian along the Mississippi coastline (30.3157° North, 89.2500° West).[19] The Louisiana domain shows **7.0 m** of surge at Pass Christian, with wind blowing from the southeast. These model results are consistent with observations. (Note that experiment NO1 represents the SRTM3 terrain as of February 2000, **before** the construction of the IHNC—Lake Borgne Surge Barrier.)

On May 19, 2005, Hassan Mashriqui, an ocean modeler at Louisiana State University's Hurricane Center, warned that the Mississippi River Gulf Outlet (MRGO) posed a danger to New Orleans because it provided a direct channel for storm surge to enter the heart of the city from the Gulf of Mexico.[27] MRGO and the Gulf Intracoastal Waterway come together at 30.0077° North, 89.9253° West to form a single east-west canal that intersects with the north-south Industrial Canal at the Turning Basin (29.991434° North, 90.019526° West) in New Orleans. The MRGO—Intracoastal confluence was called the "funnel," and this feature shows up clearly on the storm surge maps published by Dietrich et al.[28]

**Fig 15:**
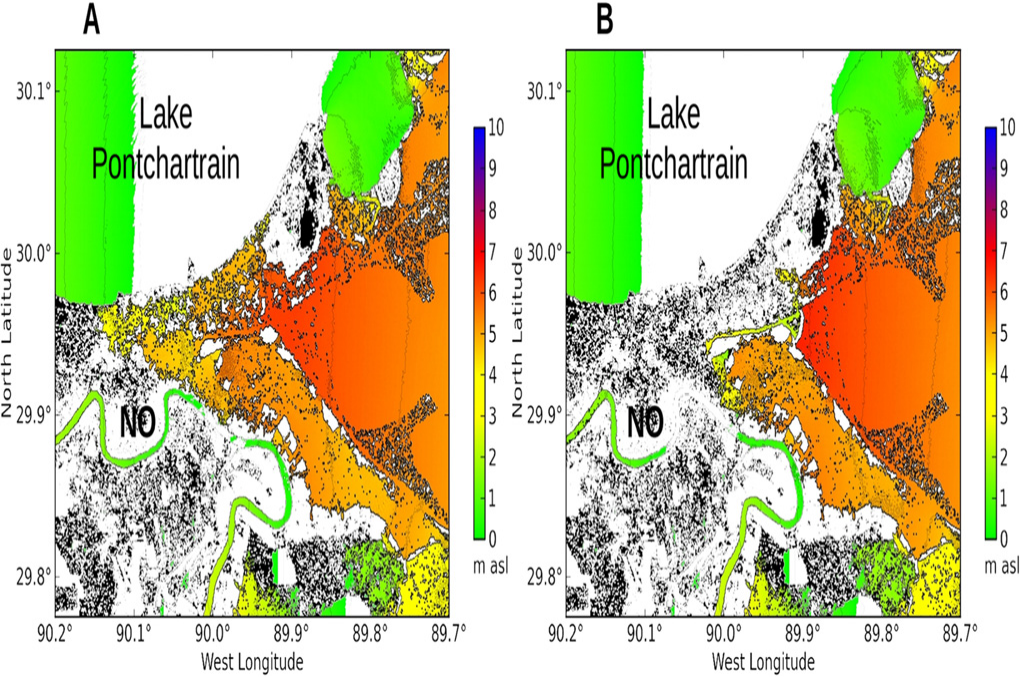
Flooding in New Orleans. Wind blows from the east. The simulation time is 15:00 hours, when the wind speed has increased to 28.75 m/s. White areas of Lake Pontchartrain have dropped below sea level. **NO** = downtown New Orleans. Panels: (A) New Orleans at the time of Hurricane Katrina 2005 (experiment NO1). (B) New Orleans after construction of the IHNC—Lake Borgne Surge Barrier in 2011 (experiment NO5).

Experiments L1, NO1, and NO5 **support** Mashriqui's prediction that the "funnel" would collect storm surge from the east and direct it toward downtown New Orleans. When winds are from the east, flooding begins at the western end of the Inner Harbor Canal (the Turning Basin) and quickly spreads along Florida Avenue to New Orleans City Park. The neck of the funnel was blocked in 2011 by construction of the Inner Harbor Navigation Canal—Lake Borgne Surge Barrier (30.0025° North, 89.9025° West) at a cost of $1.1 billion.[29] Fig 15 shows the area around the funnel before and after the surge barrier (experiment NO5). The barrier keeps Florida Avenue dry for about 5.5 extra hours (simulation time 09:30–15:00), until the surge overtops the southern arm of the funnel and water enters the city through the Lower Ninth Ward.

#### Visayas / Tacloban City

Simulation experiment V1 represents the standard directional analysis performed on the Visayas region of the Philippines, and experiment TC1 represents the corresponding analysis at Tacloban City on the island of Leyte. Storm surge at Tacloban City rises to 2.55 m above sea level when winds blow from the southeast. These experiments include neither tides nor barometric forcing.

**Fig 16:**
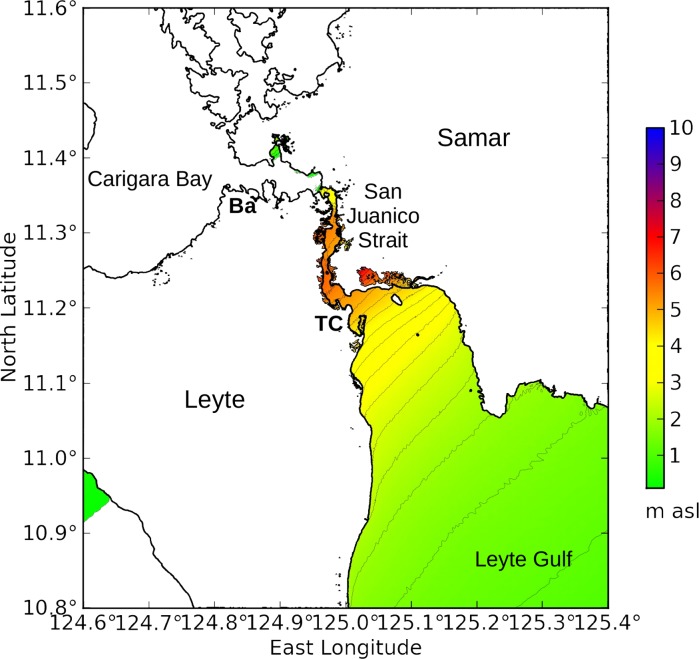
Leyte Gulf under Category 5 winds blowing from the southeast (experiment V2). The directional analysis approximates the landfall of Super Typhoon Haiyan near Tacloban City in November 2013. Colors are not shown in Carigara Bay because the sea level drops due to wind setdown. **Ba** = Babatñgon. **TC** = Tacloban City.

Simulation experiments V2 and TC2 represent the directional analysis performed with a maximum wind speed of 70 m/s, which roughly corresponds to Super Typhoon Haiyan at Category 5 in November 2013. Storm surge at Tacloban City rises to 5.0 m above sea level; news reports from shortly after the disaster reported surge heights of 6 m.[30] Fig 16 shows the modeled storm surge in Leyte Gulf. Carigara Bay experiences wind **setdown** instead of storm surge because the winds are blowing offshore, and consequently is left uncolored in the figure.

Fig 16 illustrates how two cities both close to the track of a typhoon can experience vastly different storm surge. The difference is caused by their orientation to the wind direction: Tacloban City collected the storm surge from Leyte Gulf during Typhoon Haiyan, while the waters of Carigara Bay were driven out to sea instead. Thus the **track** of an approaching hurricane, and the **orientation** of a port city relative to the expected wind direction, should be important factors in issuing surge warnings. Not all areas in the path of an approaching cyclone will experience storm surge; some coasts will experience a **drop** in water level instead.

### 3.3 Application: Tanis

Drews and Han[23] analyzed the effects of wind on a shallow strait known as the Kedua Gap, a topographical feature between two brackish lagoons in the eastern Nile delta circa 1250 BC. They named their model domain after the nearby Egyptian capital city of Tanis. The study examined the emergence of a dry land bridge resulting from wind setdown, and calculated an estimate of 4 hours for a passage across the gap. Although Drews[7] later studied the effects of wind speed and waves on the duration of the dry land bridge, no study was conducted to quantify the effects of wind **direction** at Kedua until now.

**Fig 17:**
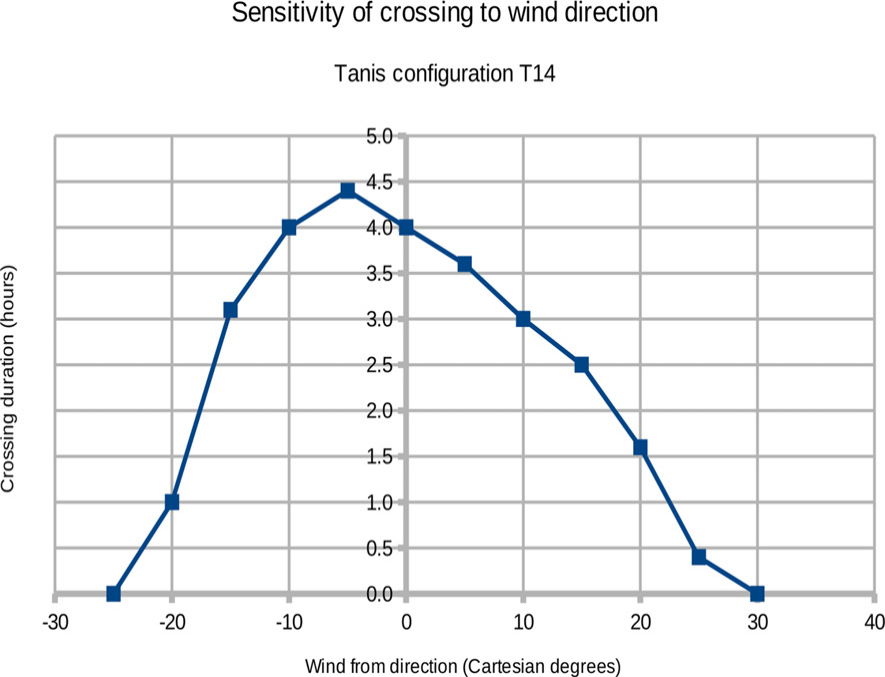
Wind direction and crossing time; model results from experiment T22. The duration of the dry passage is sensitive to the wind direction as shown. The longest crossing time occurs when the wind is blowing **from** 5° south of due east (4.4 hours). The crossing time exceeds 3.0 hours for any wind angle between -15° (south) and 10° (north).

Drews and Han[23] used a wind blowing from due east as their standard wind forcing field, and the ROMS ocean model produced a crossing time of 4.0 hours for Tanis configuration T14. The dry passage at the Kedua Gap is sensitive to the wind direction as shown in Fig 17 (experiment T22). A wind blowing from due east is not quite the optimum wind direction; the longest duration of the passage occurs when the wind is blowing from 5° south of due east. The curve in Fig 17 is not symmetric about the center line because the topography of the Kedua Gap is not symmetric. A dry crossing of the Kedua Gap requires the wind direction to vary no more than about 25°. This level of directional stability is realistic under several synoptic weather scenarios, especially those associated with strong sustained winds (the arrival of a cold front).[7]

### 3.4 Validation of 2-D, 3-D, and waves

**Fig 18:**
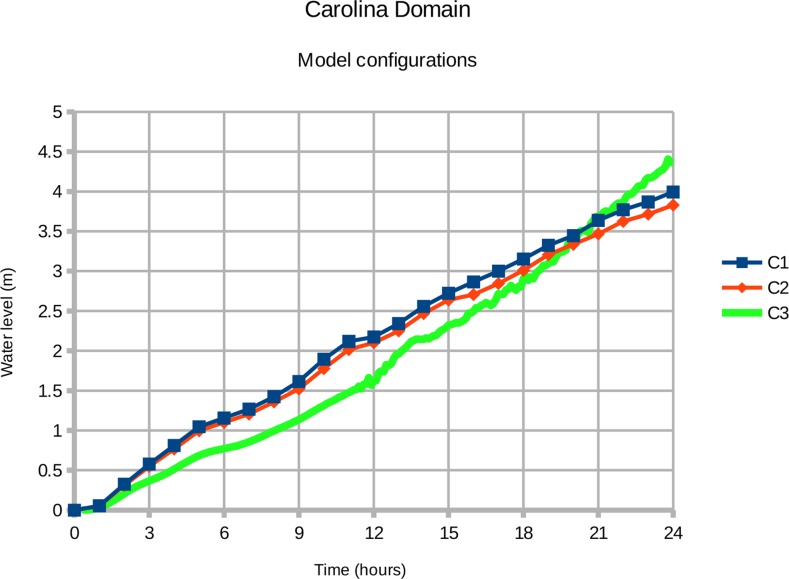
Validating the adjustment factors calculated for Lake Erie. Time series **C1** represents the COAWST ocean model configured for two-dimensional operation with an adjustment factor of 1.63. Series **C2** is COAWST in 3-D mode with an adjustment factor of 1.53. Series **C3** is COAWST in 3-D mode with waves generated by SWAN.

Experiments C2 and C3 check the adjustment factors calculated for Lake Erie against the bathymetry of the Carolina domain. C2 runs the ocean model in 3-D mode with wind blowing out of the southwest, removing the adjustment factor of 1.065. C3 runs COAWST with SWAN-generated waves, removing the adjustment factor of 1.53. The resulting water levels are shown in Fig 18.

The minor difference between time series C1 and C2 suggests that a smaller adjustment is needed here (approximately 1.023 for 2-D operation). The water levels C2 and C3 indicate that the adjustment factor of 1.53 is satisfactory overall, but the accuracy could be improved by modifying the adjustment factor according to the wind speed. Wave contribution to storm surge is not strictly linear with the square of the wind speed. We recommend running a single wind direction (on-shore) for each domain in 3-D with waves to verify that the 2-D adjustment factors are accurate.

## Discussion

ROMS / COAWST has the capability to model storm surge accurately in coastal areas ranging from the East and Gulf coasts of the United States to the Philippine Archipelago. Although the 80 m domain with an approximate span of 1° does not provide a long enough fetch for realistic storm surge, an outer 800 m domain spanning about 5° can provide boundary forcing for the inner domain. Nested grids allow the ocean modeler to examine the effects of detailed harbor features such as river channels, saltwater marshes, bridge constrictions, navigation canals, and surge barriers.

The directional analysis isolates the effect of wind direction on storm surge, and may be used to assess how vulnerable is a coastline to hurricanes and typhoons. The linear increase in wind speed over 24 hours is designed to approximate a cyclone making landfall. From a modeling standpoint, the directional analysis provides three advantages over a realistic hurricane:

1. It is much easier for an ocean modeler to blow constant wind from a fixed direction than to model a realistic hurricane making landfall. Although one could use an analytical solution to approximate a cyclonic wind field, it is more difficult to simulate the collapse of that wind field when the cyclone passes over land.[31] This problem is especially acute for significant islands like Taiwan or Luzon, where the cyclone can regain its strength after passage.
2. The directional analysis tests the domain. By blowing wind from eight compass directions, the model domain can be tested for numerical instabilities under a range of wind conditions. Even a well-chosen ensemble of landfalling hurricanes might not exercise the precise topographical condition that causes a problem during an operational forecast.
3. The directional analysis provides timing information. Surge maps that provide only the maximum envelope of water do not indicate when certain bridges or causeways will become inundated. An hourly surge profile would be useful in planning evacuations.

### 4.1 Hurricane Sandy 2012

ROMS / COAWST reproduced the observed storm surge in several locations around New York Harbor during Hurricane Sandy. The East River connection between Long Island Sound and upper New York Harbor had the effect of reducing peak surge at Kings Point by about 6% (experiments NY6 and NY9). The East River slowly drains Long Island Sound when wind is blowing from the east, and reduces the peak surge there by a small amount.

The Portal Bridge in Kearny, New Jersey (40.7533° North, 74.0950° West) protected the upper Hackensack basin from greater surge during Hurricane Sandy; the channel constriction provided by this railroad bridge delayed the storm surge by two hours from flowing toward the Meadowlands along the western spur of Interstate 95 (experiment NY6). This site would make a good candidate for a surge barrier.

### 4.2 Charleston

Charleston, South Carolina, shows a surge map that would be expected of any city situated along a straight coastline: storm surge is higher along the inland waterways than at the coast. Battery Park is located along the waterfront at the southern tip of the city of Charleston, about 7 km from the open Atlantic Ocean. Surge could rise over 4 m at Battery Park from a Category 2 hurricane.

### 4.3 Hurricane Katrina 2005

The Mississippi delta is a low-lying region extending out into the Gulf of Mexico; it is not surprising that the COAWST ocean model shows extensive inundation across the greater New Orleans area. The directional analysis reveals several aspects of the 2005 disaster:

1. Lake Pontchartrain is large enough by itself to flood Metairie and Kenner along Interstate 10 when strong winds blow from the north. The hydraulic connection through the Rigolets increases the surge from Lake Borgne, but blocking this strait would not prevent flooding.
2. Surge travels up the Mississippi River channel more rapidly than surge traveling through the bayous and broken topography of southeastern Louisiana. Those marshes protect the city of New Orleans because they have a greater effective roughness than the river channel.
3. The Mississippi River Gulf Outlet (MRGO) brought the surging waters of the Gulf rapidly into the city of New Orleans. The IHNC—Lake Borgne Surge Barrier will prevent this inflow in the future, until the surge overtops the southern bank of the canal.

Since the directional analysis can provide here a revealing **hindcast** for New Orleans and Hurricane Katrina 2005, the same analysis can likely produce a useful **forecast** for coastal cities that have not yet suffered a devastating hurricane.

### 4.4 Typhoon Haiyan 2013

The directional analysis would have shown the vulnerability of Tacloban City to a typhoon approaching on a track similar to Typhoon Haiyan. Tacloban City is located at the northwestern end of Leyte Gulf, and this relatively shallow body of water collects storm surge and focuses it toward San Juanico Strait.

### 4.5 Surge maps from the National Hurricane Center

The National Hurricane Center has produced surge maps of the coastal United States since about 1990; those maps use a combination of storm configurations and tracks to estimate the Maximum Envelope Of Water (MEOW).[32] That worst-case approach means that most of the coast will be over-estimated during an actual event, surge will fall short of the MEOWs, and some citizens may respond by ignoring the warnings.[33] The process of combining multiple tracks into a single surge map inevitably loses valuable track information. For example, the NHC surge maps do not distinguish between Charleston receiving a left-side strike from a hurricane and a right-side strike. The accuracy of surge maps can be improved by taking the projected hurricane track into account. Wind-driven surge is caused by **onshore** winds, not the **offshore** winds that occur on the left side of a North Atlantic hurricane.

In 2014 the National Hurricane Center introduced a new set of detailed maps that project storm surge flooding.[34] These maps will be issued in conjunction with a forecast track and intensity. The new forecast product should reduce the over-estimation problem. A detailed set of maps derived from the directional analysis would serve as a useful supplement to the new surge maps from the NHC. Because the directional maps are pre-calculated independently of hurricane track, they could be prepared and published well before the next hurricane/typhoon season. Civil authorities could assess and reduce the vulnerability of their coastlines, and trained emergency managers could make preliminary plans even while the projected track error is large.

The directional analysis uses winds of Category 2 speed for the standard configuration. Researchers could expand the analysis by including a range of wind speeds, as we have done with Typhoon Haiyan 2013 by ramping up the wind speed to Category 5. By including Categories 1–5, the directional analysis would provide a more comprehensive picture of the potential for storm surge over a range of meteorological conditions.

## Conclusions

The adjustments to ROMS / COAWST calculated for Lake Erie[7] are also valid for Hurricane Sandy 2012. COAWST accurately reproduces the observed surge height and timing in New York Harbor. Nested grids allow for detailed modeling of harbor features combined with a long fetch required for accurately modeling surge heights. COAWST's algorithm for wetting and drying provides an effective way to model inland flooding.

The directional analysis calculates the potential for storm surge based on wind direction. For example, Wilmington, Delaware could experience surge heights over 8 m if a Category 2 hurricane were to make landfall in coastal Maryland. The analysis also provides some timing information for an approaching cyclone. Surge maps can be prepared for the world's coastlines that are within the latitude bands of tropical cyclones. These maps can be used in advance to prepare the population and infrastructure, and during the cyclone event when combined with a track forecast.

Surge maps are also applicable to any coastal area that may experience high onshore winds, such as the Irish Sea and the Elbe Estuary at Hamburg, Germany.[7] The directional analysis is an ocean modeling product that can assist coastal communities in protecting their citizens and property.

## Supporting Information

S1 File Google Earth overlay (KML). NewYorkHarbor.xml contains modifications to the terrain of New York Harbor to permit current flow through river channels.
(XML)
